# Impact of a simplified *in situ* protocol on enamel loss after erosive challenge

**DOI:** 10.1371/journal.pone.0196557

**Published:** 2018-05-07

**Authors:** Natália Mello Santos, Maísa Camillo Jordão, Franciny Querobim Ionta, Fernanda Lyrio Mendonça, Camilla Cristina Lira Di Leone, Marília Afonso Rabelo Buzalaf, Thais Marchini Oliveira, Heitor Marques Honório, Thiago Cruvinel, Daniela Rios

**Affiliations:** 1 Department of Pediatric Dentistry, Orthodontics and Public Health, Bauru School of Dentistry, University of São Paulo, Bauru, São Paulo, Brazil; 2 Department of Biological Sciences, Bauru School of Dentistry, University of São Paulo, Bauru, São Paulo, Brazil; University of Washington, UNITED STATES

## Abstract

This study investigated the effect of the period of use and location of intraoral appliances on enamel surface loss. This randomized, single blind *in situ* study was conducted in 2 crossover phases based on the period of use, in which maxillary and mandibular appliances were simultaneously worn. Bovine enamel blocks (n = 120) were randomly divided among the studied groups by surface hardness. In each phase, fifteen volunteers used one maxillary appliance and two mandibular appliances for 5 days. Erosive challenge was performed 4X/day by immersion in 0.01 M HCL for 2 minutes. In the continuous phase, the intraoral appliances were worn for 20 hours. In the intermittent phase the appliances were worn for 8 hours and 30 minutes. Enamel loss was determined profilometrically. The discomfort of use of the appliances were evaluated in a questionnaire. Data were analyzed by two-way ANOVA/Tukey’s test and chi-square test (p<0.05). The maxillary appliance promoted higher enamel loss compared to the mandibular one (p<0.001). Intermittent use of appliances resulted in similar enamel loss to the continuous one (p = 0.686). All volunteers preferred to use the maxillary appliance in an intermittent regimen. The intermittent use of maxillary appliance is a simplified reliable protocol appropriated for *in situ* erosion studies in enamel.

## Introduction

The high prevalence of erosive dental wear has increased clinical concern [1,2] and investigations for anti-erosive agents are ongoing [3,4]. As randomized clinical trials do not allow precise measurement of tooth tissue loss, agents and therapies have been tested mostly through *in vitro* and *in situ* studies [5,6]. *In situ* studies, the substrate is exposed to the oral environment and acid type and exposure time can be standardized [6,7]. In addition, variables can be tested individually and new variables can be introduced gradually. Different *in situ* erosion experimental models have been reported [6,8,9]. Adequate models should simulate the clinical situation as close as possible, especially concerning the effect of human saliva, which is known to be a relevant factor in the development and progression of erosive lesions [10,11,12].

*In situ* models can be conducted with fixed or removable intra-oral appliances [6]. Removable appliances can be either of intermittent use, in which a volunteer uses the appliance only during work hours [13,14] or continuous use during day and night. The advantage of the intermittent appliance is the possible supervision of the protocol, increasing volunteers’ compliance. A previous *in situ* study has shown that a 12-hour overnight use of the intraoral appliance resulted in a similar erosion prevention compared to a 2-hour daytime use [15]. In addition, the use of an intraoral appliance during sleep did not improve saliva ability to reharden an erosion lesion when compared to a 2-hour daytime use [15,16]. However, no study has clarified the impact of overnight salivary effect on enamel loss, considering both demineralization and remineralization episodes using an *in situ* experimental erosive cycling model.

Another aspect that varies among *in situ* protocols is the location of the intraoral appliances, with most of them positioned in the maxilla or in the lower buccal region [6,17]. Depending on the position, the appliance will be in contact with saliva from different glands, affecting the level of erosion [18]. Moreover, a recent study has shown that the protein profile of the acquired pellicle changes according to its location in the dental arches, which might influence the protective ability of the protein film against erosion [19]. In previous studies involving initial erosive lesions, the location of the intraoral appliance did not interfere in the rehardening potential of saliva on enamel [16] and its ability to protect against initial erosive demineralization [15]. However, no information is available considering erosive cycles and advanced erosive lesions.

The discomfort and burden caused by the use of an intraoral appliance can reduce compliance in *in situ* experimental procedures, impairing the reliability of results. A simplified protocol and a comfortable appliance might result in a higher commitment during the experiment. However, there is no information related to volunteer experience of using intraoral appliances.

The aim of this *in situ* study was to investigate the effect of the period of use of intraoral appliances on enamel surface loss when considering erosive cycling. The research questions were: (1) does the use of the appliance during work hours lead to similar enamel loss when compared to its continuous use, despite the absence of the overnight protective effect of saliva? (2) does the location of the appliance (maxillary or mandibular) play a role on the degree of enamel loss when enamel blocks are subjected to *in situ* erosive challenges? The hypotheses tested were that (1) *in situ* erosion protocols with intermittent use or continuous use of intraoral appliance result in similar enamel loss; and (2) the use of maxillary or mandibular removable appliance promote similar enamel loss. The volunteers comfort and preference regarding the protocols were also evaluated.

## Materials and methods

### Experimental design

**Fig 1:**
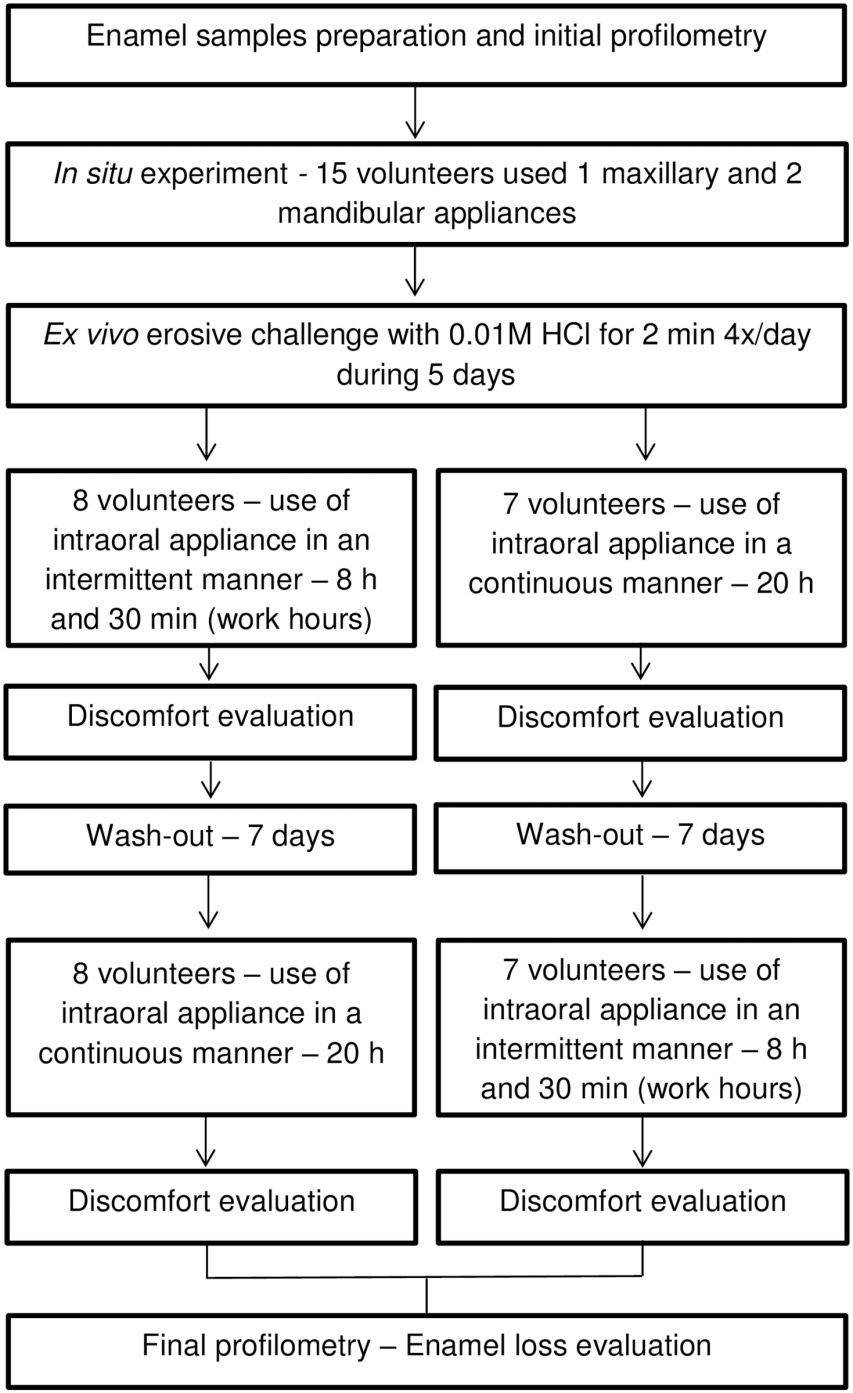
Flowchart of the study.

This study was conducted in accordance with the guidelines of good clinical practice and the Declaration of Helsinki. Ethical approval was granted by the local Ethics Committee (protocol n° CAAE 24216514.8.0000.5417). The subjects were trained for the required procedures (before the starting the *in situ* experiment, the volunteers wore the appliances without the enamel blocks and performed a day of erosive cycling) and received written instructions and a printed schedule. Informed consent was obtained before starting the study. The study followed a single-blind, randomized, crossover protocol comprising 2 phases of 5 days each, with an interval of 7 days between them. The factors under study were type of appliance (mandibular or maxillary) and period of use (continuous or intermittent). Fifteen volunteers used the mandibular and maxillary appliances simultaneously and, in each phase, a specific period of use was tested. Bovine enamel blocks were selected by surface hardness (n = 120) and randomly divided among the study groups. The erosive challenge was performed *ex vivo* by immersing the intraoral appliances in hydrochloric acid solution for 2 minutes, 4 times a day for 5 days. The discomfort caused by the use of the appliances was evaluated by a questionnaire and enamel loss was assessed using profilometry (Fig 1).

### Enamel samples preparation

Enamel slabs were prepared from bovine teeth obtained from Mondelli Food Industry S.A. (Bauru, São Paulo, Brazil). Slabs (4×4×3 mm^3^) were cut using a cutting machine (Isomet Low Speed Saw, Buehler Ltd., Lake Bluff, IL, USA) and two diamond disks (Extec Corp., Enfield, CT, USA) separated by a 4-mm spacer. The block surface was ground flat with water-cooled abrasive discs (320, 600 and 1200 grades of Al_2_O_3_ papers; Buehler Ltd., Lake Bluff, IL, USA) and polished with wet felt paper and diamond suspension (1 μm; Buehler Ltd., Lake Bluff, IL, USA). Between the polishing steps, the enamel blocks were cleaned in an ultrasonic device with deionized water for 10 min (T7 Thornton, Unique Ltda., São Paulo, SP). The surface hardness was determined by performing five indentations at 100 μm distances from each other in the center of the blocks (Knoop diamond, 25 g, 10s, Hardness tester from Buehler, USA). Specimens with hardness values 10% lower or higher than the overall mean were excluded from the study. One hundred and twenty enamel blocks with average initial surface hardness of 339±14 KPa/mm^2^ were selected and randomly assigned to the study phases, appliances, and volunteers. The enamel blocks were sterilized by exposure to ethylene oxide gas previously to the *in situ* phases [20].

### Initial profilometry

Enamel slabs were marked with a scalpel blade (Embramac, Itapira, SP, Brazil) to define 1.0-mm control areas (at the border) and 2.0-mm test area (at the center). One of the control areas was marked with a 1/4 drill to assure that final and initial profiles were measured at exactly the same sites. The initial profile of the enamel blocks was evaluated by a profilometer (Marh, MarSurf GD 25, Göttingen, Germany) coupled to a computer with a contour software (MarSurf XCR 20). Slabs were fixed to a special holder and the first profile was measured at the identification mark; the slab location was recorded [21]. Subsequently, for each block, four readings were made at the following distances from the mark over the y-axis: 0.5; 0.75; 1.0 and 1.25 μm. The profile of each reading was individually saved.

The two borders of the enamel blocks (1.0 mm each) were protected with cosmetic nail varnish (Maybelline Colorama, Cosbra Cosmetics Ltda, São Paulo, SP, Brazil) and served as reference areas (no acid exposure during *in situ* phase) for enamel loss measurement.

### Volunteer selection and *in situ* experiment

Fifteen healthy adult volunteers (14 female, 1 male, aged 19–26 years) took part of the study. The inclusion criteria were residing in the same fluoridated area (0.70 mg F/L), stimulated physiological salivary flow rate >1 mL/min, non-stimulated physiological salivary flow rate >0.25 mL/min, and adequate oral health (without caries and erosive lesions or significant gingivitis/periodontitis evaluated through rigorous clinical examination based on the ICDAS and BEWE scores) [22,23]. The exclusion criteria were systemic diseases, pregnancy or breastfeeding, current orthodontic treatment, fluoride compounds use in the last two months, and smoking.

Sample size calculation was based on a pilot *in situ* study with 3 volunteers. A sample size of 12 volunteers was estimated based on an α-error of 5%, β-error of 20%, 0.69 μm estimated standard deviation, and 1 μm minimum detectable difference in means. To account for possible dropouts, 15 volunteers were selected.

**Fig 2:**
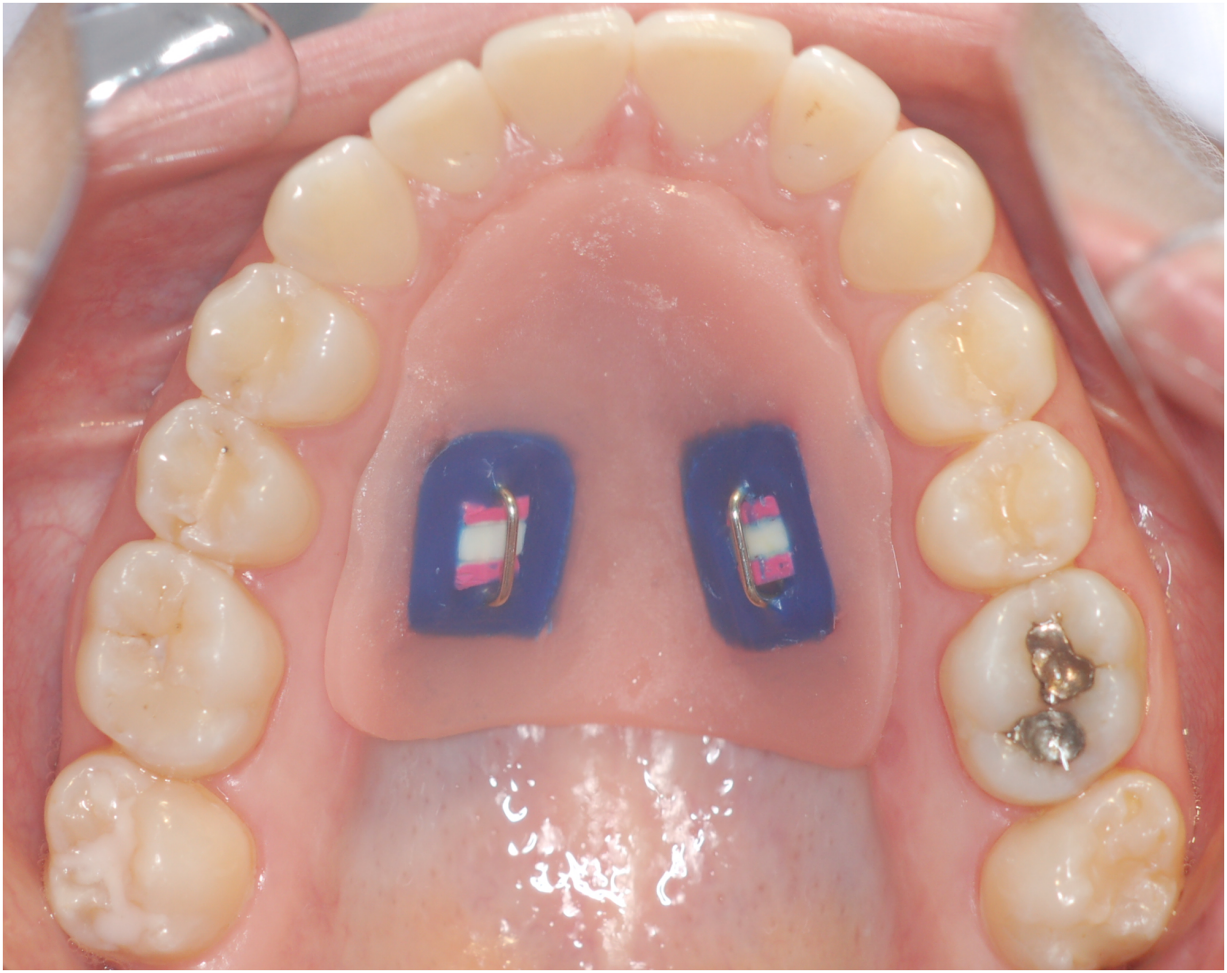
Volunteer wearing maxillary intraoral appliance.

**Fig 3:**
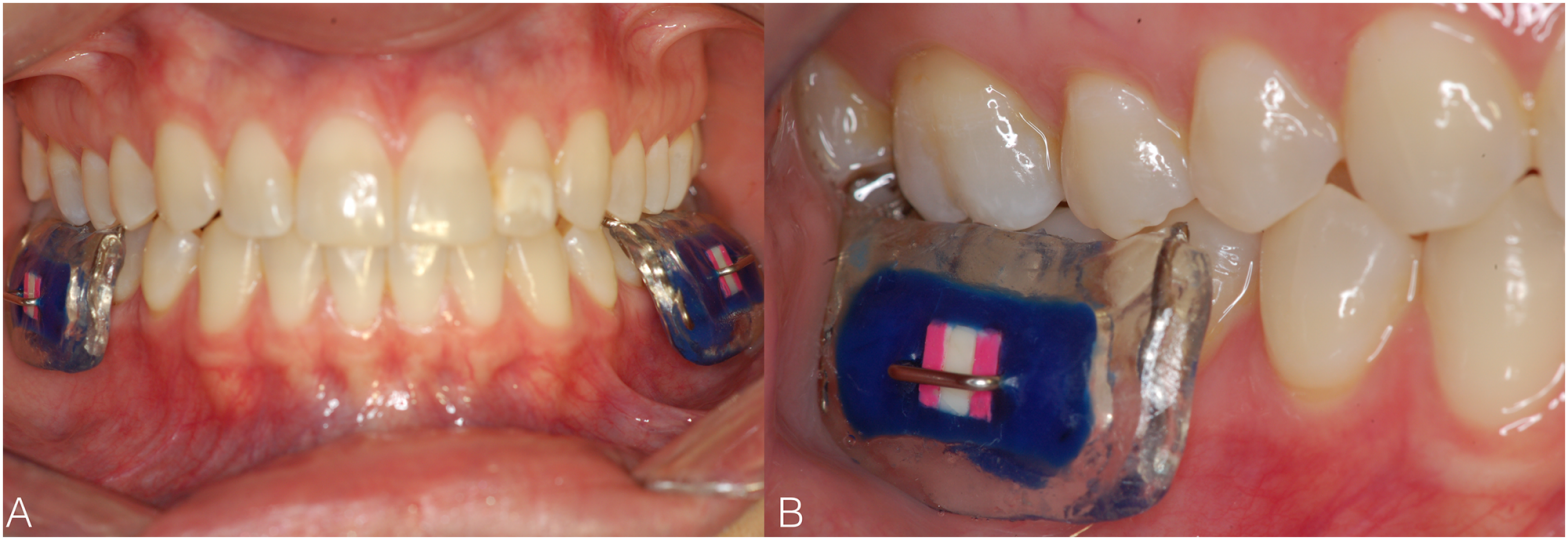
Volunteer wearing mandibular intraoral appliances (A). Approximate view of the appliance (B).

The maxillary and mandibular intraoral appliances were made with acrylic resin on upper and lower arch plaster models of each volunteer. The maxillary appliance had two vertical rows, on the right and left sides, with a cavity (5×5×3 mm^3^) in each for enamel slabs fixation (two slabs per appliance) (Fig 2). For the mandible, an appliance was made for each side of the arch, with a row and cavity for enamel slabs fixation (one slab per appliance). The appliances were fixed to the first permanent molars by Adams clasp, thus preventing displacement, as described previously [6,24] (Fig 3). The enamel slabs were fixed to the appliances using wax. An orthodontic wire passing over the enamel blocks without touching them, fixed to the cavities, was used to prevent abrasion by the tongue or oral mucosa.

Seven days prior to and during the experimental period, the volunteers brushed their teeth with a standardized toothbrush (Curaprox 5460 ultra soft, Curaden Swiss, Switzerland) and fluoride toothpaste (Triple Action, 1.450 ppm F, Colgate, Brazil). The volunteers were asked not to use other fluoride products.

The intraoral appliances were worn by the volunteers in the evening prior to the day of the experiment initiation just after their last oral hygiene to allow the formation of the acquired pellicle. The erosive regimen began the next day. Seven volunteers started the *in situ* phase using the intraoral appliance in a continuous manner and the other 8 volunteers in an intermittent manner. For the continuous use, the maxillary and mandibular intraoral appliances were worn simultaneously, during day and night (20 h/day) for 5 days and stored in wet gauze during main meals and oral hygiene (four times daily, 1 h each, total of 4 h). In the intermittent use, the volunteers wore the intraoral appliances simultaneously during work hours (7:45 am until 6 pm) for 5 days. The appliances were removed for 1 h 45 min for meals (total of 8 hours and 30 minutes of daily use). When not in use, the appliances were stored in wet gauze and refrigerated to prevent dehydration of the enamel slabs. On both groups, the erosive challenges were performed four times a day (8 am, 10 am, 2 pm and 4 pm) by *ex vivo* immersion of the appliances containing the enamel slabs into 150 mL of 0.01 M hydrochloric acid pH 2.3 at room temperature (37°C) for 2 min [25,26]. Immediately after erosion, the appliances were washed in tap water and reinserted into the mouth.

### Oral appliance discomfort evaluation

At the beginning of each *in situ* phase, the volunteers received a questionnaire regarding use and speech discomfort, such as pain during and after removal of the appliance (S1–S4 Files). The answers were dichotomized into yes or no. The last question asked about the volunteer preference regarding type of appliance and period of use.

### Final profilometry

After the *in situ* phases, the enamel slabs were removed from the intraoral appliances and the cosmetic nail varnish was removed from the surface using the tip of a scalpel blade positioned in the angle between the base and external wall of the block. Enamel slabs were fixed in the holder on the profilometer according to its initial measurement. The identification mark was used to confirm the reproducibility of the position. Four readings were performed using the same software (XCR 20, MarSurf GD 25, Göttingen, Germany) and measurement parameters described above (*Initial profilometry)*.

**Fig 4:**
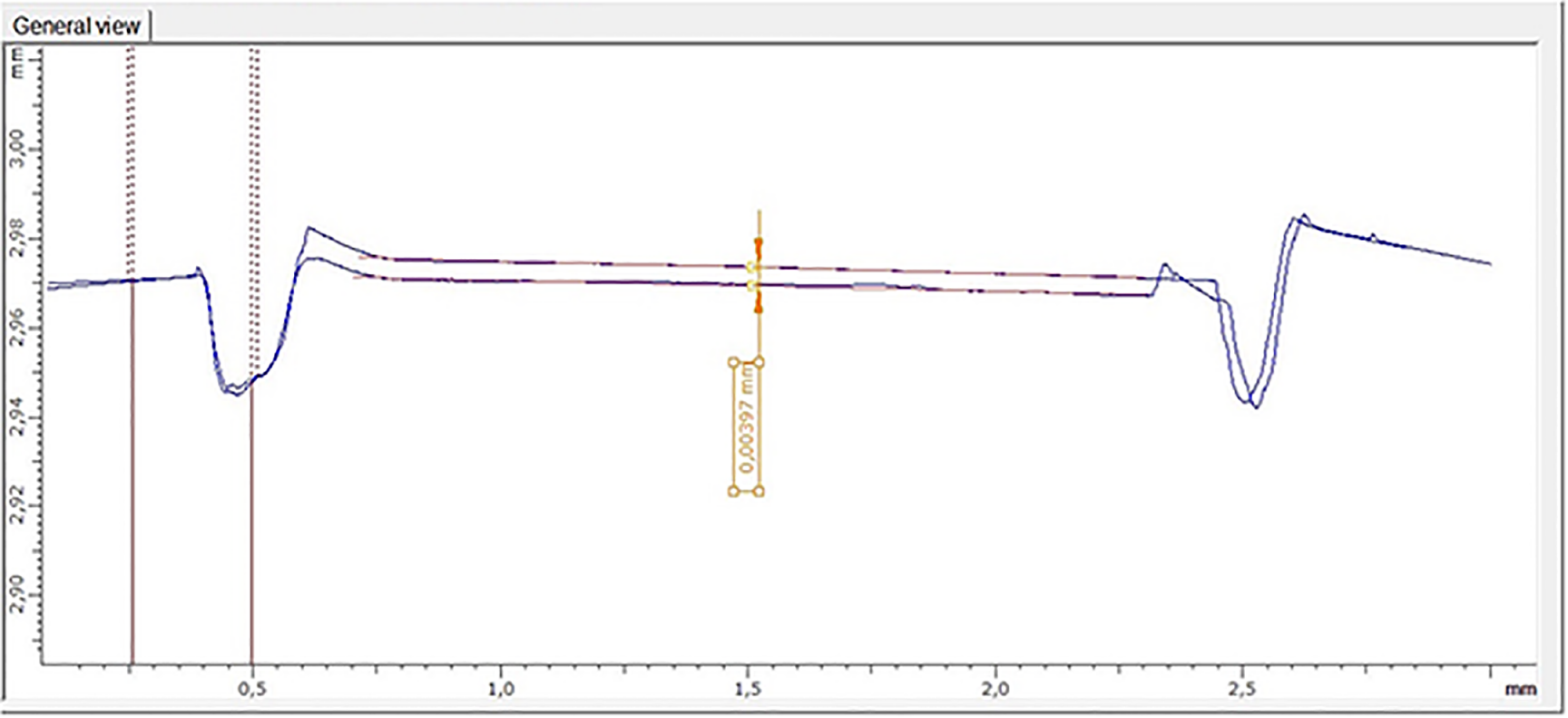
Superimposition of Initial and final profiles (in blue). Parallel regression lines were constructed with a 0.5 mm length on the initial and final profile (in red). The vertical distance between the regression lines was defined as the amount of tissue loss (μm).

Initial and final profiles were superimposed for each of the four graphs. Parallel regression lines were constructed with a 0.5 mm length on the initial and final profile. The vertical distance between the regression lines was defined as the amount of tissue loss (μm) (Fig 4). Tissue loss was expressed as the mean values of four superimposed graphs.

### Statistical analysis

Statistical analysis was performed with the STATISTICA software version 10.0 (StatSoft Inc., Tulsa, Okla., USA). The assumptions of equality of variances and normal distribution of errors were checked. Since the assumptions were met, appropriate ANOVA models for the analysis of crossover data was used to compare the effects of period of use (continuous or intermittent), type of appliance (mandibular or maxillary), study period and study sequence, and their interactions on enamel loss (response variable). The models also included a random effect to correlate multiple measurements within a subject. Tukey’s test was applied for multiple comparisons and the level of significance was set at 5%. The association between maxillary/mandibular appliance and presence of discomfort or pain, and between intermittent / continuous use and presence of discomfort or pain were analyzed by chi-square test.

## Results

All volunteers completed the *in situ* phase and answered the questionnaire according to the protocol. Table 1 shows the mean enamel loss of each experimental group. Statistical analysis showed no influence of study period and study sequence on the results. The location of the intraoral appliance influenced the overall enamel loss (p < 0.001) and there was no interaction between the factors studied (p = 0.561). The enamel slabs placed in the maxillary appliance presented significantly higher erosive enamel loss when compared to the enamel slabs of the mandibular appliance. The intermittent use of the appliances resulted in similar enamel loss when compared to the continuous use (p = 0.686).

**Table 1:** Means and standard deviation (SD) values of enamel loss (μm) after intermittent or continuous use of mandibular or maxillary appliances.

Groups	Maxillary Appliance (μm)	Mandibular Appliance (μm)
**Intermittent use**	2.30 (± 0.81) ^a^	1.67 (± 0.68)^b^
**Continuous use**	2.44 (± 0.78)^a^	1.65 (± 0.64)^b^

Values followed by distinct letters differ significantly (two way ANOVA and Tukey’s Test, p < 0.05).

Table 2 describes the discomfort reported by the volunteers when the mandibular and maxillary appliances were worn intermittently or continuously. The statistical analysis showed that discomfort regarding speech and use, pain during use and after the appliance removal as well as discomfort of five-day use were significantly associated to the mandibular appliance. None of the above-mentioned factors were significantly associated to the intermittent or continuous use of the appliances. All the volunteers chose the maxillary appliance used intermittently as preferred protocol.

**Table 2:** Discomfort and pain (%) reported by the volunteers (n = 15) when mandibular and maxillary appliances were worn intermittently or continuously.

Questionnaire	Variable	No	Yes	P value
**Discomfort on speech**	Mandibular	26.7%	73.3%	<0.001
	Maxillary	80%	20%	
	Intermittent	53.3%	46.7%	0.79
	Continuous	53.3%	46.7%	
**Discomfort on use**	Mandibular	30%	70%	<0.001
	Maxillary	86.7%	13.3%	
	Intermittent	51.4%	48.6%	0.79
	Continuous	48%	52%	
**Pain on use**	Mandibular	56.7%	43.3%	<0.001
	Maxillary	100%	0%	
	Intermittent	83.3%	16.7%	0.53
	Continuous	73.3%	26.7%	
**Pain after appliance removal**	Mandibular	46.7%	53.3%	<0.001
	Maxillary	100%	0%	
	Intermittent	76.7%	23.3%	0.77
	Continuous	70%	30%	
**Discomfort on use for 5 days**	Mandibular	26.7%	73.3%	<0.001
	Maxillary	93.3%	6.7%	
	Intermittent	60%	40%	0.79
	Continuous	60%	40%	

p < 0.05 indicates significant associations between variables (chi-square test).

## Discussion

Understanding the factors that can influence outcomes of *in situ* studies regarding enamel erosion may guide future research, diminishing variability among studies. Based on our results the first hypothesis was accepted. Enamel blocks subjected to an *in situ* protocol of erosion in an intraoral appliance used intermittently showed similar surface loss compared to the blocks subjected to continuous use. The oral cavity presents hemostatic mechanisms to preserve tooth tissues [12,27] that are mainly attributed to the effect of the saliva. Saliva’s flow rate and inorganic/organic constituents are able to dilute, clear, neutralize and buffer acidic substances, enhance enamel mineral deposition by providing calcium, phosphate and fluoride, repair the initial erosive lesion, and reduce erosive demineralization by the formation of the acquired pellicle [12,28]. In the present study, the duration difference between the continuous and intermittent protocols was of 11 hours and 30 minutes per day, mainly overnight, and this did not play a role on the protective and reparative effect of saliva against erosion. A possible explanation for this result is the low salivary flow rate during sleep that provided little protective effect against erosion, since all the properties of saliva depend on salivary flow [29]. In addition, the interplay among enamel, saliva, and erosive acid at the distinct demineralization and remineralization phases should be considered [15,16]. Mendonça et al. tested *in situ* the effect of enamel salivary exposure time prior to an acid challenge on demineralization prevention (initial erosion) [15]. The authors found that 2 hours of *in situ* exposure to saliva improves enamel protection against initial erosive lesion development similarly to 30-minute and 1-hour exposures [15]. On the other hand, keeping the blocks in saliva for a 12-h overnight period did not enhance this protection [15]. In relation to remineralization, Alencar et al. (2016) assessed the effect of different salivary exposure times on the rehardening of acid-softened enamel and found that the use of an intraoral appliance for 10 hours during sleep did not improve the enamel rehardening from the initial erosion lesion [16]. Although the term remineralization is generally used in dental erosion, the mineral uptake is confined to the surface and near-surface softened layer [30], differently from what occurs with caries. In the current study, the interval between each erosive challenge was set at two hours for the intermittent and continuous protocols to guarantee the protective effect against erosion and rehardening action of saliva.

The location of the intraoral appliance was found to influence enamel surface loss when considering erosive cycling, and thus, the second study hypothesis was rejected. The blocks located in the maxillary appliances showed higher enamel loss when compared to the blocks in the lower buccal area. Salivary glands located in different sites of the oral cavity render different characteristics of flow rate and acquired enamel pellicle (AEP) formation. Removable appliances trigger a mechanical stimulus, increasing saliva production of the parotid gland, which is responsible for the buffering capacity and the increase of calcium and phosphate ions [31,32,33]. Therefore, the blocks near the parotid glands would be less susceptible to acid attack, as saliva’s buffering effect would lead to a fast pH recovery and enamel rehardening [12]. However, in the present study, the erosive challenge was performed outside the mouth for better protocol standardization and to prevent acid exposure of the volunteers’ natural teeth [34]. This approach cannot simulate adequately the buffering and acid dilution effects of saliva [11,12]. Thus, the blocks in the lower buccal area of the second premolar and first permanent molar(mandibular appliance), which are close to the parotid glands, might have benefitted from rehardening but not from pH recovery effects from saliva.

During enamel surface exposition to the oral environment an organic film known as AEP is formed [11]. The AEP provides partial protection against erosion [35,36,37], which is influenced by AEP composition and thickness [12] that varies according to the incidence of mechanical forces and the salivary gland. The AEP protein composition also changes according with its location in the dental arches [19]. Non-stimulated salivary flow has high concentration of mucin, which is the major structural constituent of the pellicle [38]. In a recent study, the AEP treated with fluoride and tin provided the best protection against erosion and mucins were identified in higher proportion [39]. The submandibular gland contributes predominantly to the non-stimulated saliva secretion, which is mucin-rich. This may explain the higher resistance against erosion of the mandibular blocks, resulting in less enamel loss.

The softened erosive layer consists of a group of crystals separated by large spaces [40], which are vulnerable to abrasive or frictional forces [27] causing further enamel loss. Seong et al. (2017) demonstrated that enamel loss resulting from tongue abrasion on eroded surfaces could be an unavoidable consequence of oral function [9]. Therefore, the higher enamel loss found in the blocks located in the maxillary appliance could be a partial result of tongue abrasion over the eroded surfaces, despite the metal wires placed over the enamel blocks to avoid the abrasive effect of soft tissues and tongue. Previous studies also used different methods to avoid the influence of abrasion in the samples such as the placement of retaining wires [15] or sample fixation 0.5 mm below the contact surface; however there are studies that did not protect the sample from influence of mechanical forces [13,16,17]. Therefore, in future studies, the impact of the appliance location on enamel loss should be evaluated considering the effect of these forces.

The period of use and the location of intraoral appliances were also investigated according to the volunteers’ point of view. All volunteers preferred to use the intraoral appliance during the day. They reported that this protocol was easier to comply with. However, there was no difference in speech and use discomfort, as well as pain during and after the appliance removal between the intermittent and continuous use. This result shows that although used for a shorter period, the intermittent protocol does not totally avoid discomfort. Meanwhile, the volunteers reported more discomfort and pain for the mandibular appliance, and all preferred the maxillary appliance. It is important to emphasize, however, that the maxillary and mandibular appliances were used at the same time and probably some overlapping of discomfort sensations occurred. Therefore, additional studies are needed to investigate volunteer experiences using intraoral appliances.

In conclusion, erosive *in situ* protocols using intermittent intraoral appliances resulted in similar enamel loss compared to continuous appliances. The use of the maxillary appliance promoted higher enamel loss than the mandibular one. All volunteers preferred the maxillary appliance used during the day. Therefore, the intermittent use of a maxillary appliance is a simplified reliable protocol appropriated for *in situ* erosion studies in enamel. This is the first step to standardize *in situ* models, and in future studies, the influence of other variables on treatments response should be evaluated.

## Supporting information

S1 File Questionnaire of the continuous phase. (DOCX)

S2 File Questionnaire of the intermittent phase. (DOCX)

S3 File Questionnaire of the continuous phase in Portuguese. (DOCX)

S4 File Questionnaire of the intermittent phase in Portuguese. (DOCX)
