# Supplementary material for: Impact of a simplified in situ protocol on enamel loss after erosive challenge
Source: PLoS One. 2018 May 7;13(5):e0196557. doi: 10.1371/journal.pone.0196557 (PMC5937767; doi:10.1371/journal.pone.0196557)
Supplement: S1 File — (DOCX) [file pone.0196557.s001.docx]

**Questionnaire regarding the use of mandibular and maxillary appliances in in situ studies**

**In relation to comfort: WHEN USED 24h/day - continuous**

1. Has the appliance disrupted your speech?

Mandibular appliance Maxillary appliance

Yes ( ) No ( ) Yes ( ) No ( )

2. Did the appliance cause discomfort during sleep?

Mandibular appliance Maxillary appliance

Yes ( ) No ( ) Yes ( ) No ( )

3. Did the appliance cause discomfort during the day when you were at rest?

Mandibular appliance Maxillary appliance

Yes ( ) No ( ) Yes ( ) No ( )

**In relation to pain:**

1. Did the appliance cause pain during daytime use?

Mandibular appliance Maxillary appliance

Yes ( ) No ( ) Yes ( ) No ( )

2. Did the appliance cause pain during nighttime use?

Mandibular appliance Maxillary appliance

Yes ( ) No ( ) Yes ( ) No ( )

3. After removing the appliance did you feel pain?

Mandibular appliance Maxillary appliance

Yes ( ) No ( ) Yes ( ) No ( )

4. If so, did this pain last how long after the end of in situ phase?

Mandibular appliance Maxillary appliance

1 day ( ) 2 days ( ) More than 2 days ( ) 1 day ( ) 2 days ( ) More than 2 days ( )

If you need to use one of these appliances again, which one would you prefer?

Mandibular appliance ( ) Maxillary appliance ( )
