# Supplementary material for: Impact of a simplified in situ protocol on enamel loss after erosive challenge
Source: PLoS One. 2018 May 7;13(5):e0196557. doi: 10.1371/journal.pone.0196557 (PMC5937767; doi:10.1371/journal.pone.0196557)
Supplement: S3 File — (DOCX) [file pone.0196557.s003.docx]

**Questionário referente ao uso de dispositivos palatinos e mandibulares em estudos *in situ***

**Em relação ao conforto: SEMANA EM QUE USOU 24H/DIA – contínuo**

1. O dispositivo atrapalhou sua fala?

Dispositivo palatino Dispositivo mandibular

Sim ( ) Não ( ) Sim ( ) Não ( )

2. O dispositivo causou desconforto durante o sono?

Dispositivo palatino Dispositivo mandibular

Sim ( ) Não ( ) Sim ( ) Não ( )

3. O dispositivo ocasionou desconforto durante o dia nos momentos em que você estava em repouso?

Dispositivo palatino Dispositivo mandibular

Sim ( ) Não ( ) Sim ( ) Não ( )

**Em relação à dor:**

1. O dispositivo provocou dor durante o uso diurno?

Dispositivo palatino Dispositivo mandibular

Sim ( ) Não ( ) Sim ( ) Não ( )

2. O dispositivo provocou dor durante o uso noturno?

Dispositivo palatino Dispositivo mandibular

Sim ( ) Não ( ) Sim ( ) Não ( )

3. Após a remoção do dispositivo você sentiu dor?

Dispositivo palatino Dispositivo mandibular

Sim ( ) Não ( ) Sim ( ) Não ( )

4. Se sim, esta dor permaneceu quanto tempo após o término da fase in situ?

Dispositivo palatino Dispositivo mandibular

1 dia ( ) 2 dias ( ) Mais de 2 dias ( ) 1 dia ( ) 2 dias ( ) Mais de 2 dias ( )

Caso você precise utilizar um desses dispositivos novamente, qual deles você preferiria?

Dispositivo palatino ( ) Dispositivo mandibular ( )
